# High-Dynamic-Range Integrated NV Magnetometers

**DOI:** 10.3390/mi15050662

**Published:** 2024-05-18

**Authors:** Tianning Wang, Zhenhua Liu, Yankang Liu, Bo Wang, Yuanyuan Shen, Li Qin

**Affiliations:** 1 State Key Laboratory of Dynamic Measurement Technology, North University of China, Taiyuan 030051, China; 13845291760@163.com (T.W.); nuistlzh@163.com (Z.L.); 17633571257@163.com (Y.L.); brucewang9119@163.com (B.W.); 15534064518@163.com (Y.S.); 2 School of Instrument and Electronics, North University of China, Taiyuan 030051, China; 3 School of Semiconductor and Physics, North University of China, Taiyuan 030051, China

**Keywords:** diamond NV centers, integrated magnetometer, high dynamic range, magnetic field measurement

## Abstract

High-dynamic-range integrated magnetometers demonstrate extensive potential applications in fields involving complex and changing magnetic fields. Among them, Diamond Nitrogen Vacancy Color Core Magnetometer has outstanding performance in wide-range and high-precision magnetic field measurement based on its inherent high spatial resolution, high sensitivity and other characteristics. Therefore, an innovative frequency-tracking scheme is proposed in this study, which continuously monitors the resonant frequency shift of the NV color center induced by a time-varying magnetic field and feeds it back to the microwave source. This scheme successfully expands the dynamic range to 6.4 mT, approximately 34 times the intrinsic dynamic range of the diamond nitrogen-vacancy (NV) center. Additionally, it achieves efficient detection of rapidly changing magnetic field signals at a rate of 0.038 T/s.

## 1. Introduction

At present, magnetic field measurements have significant applications in various fields. Therefore, rapid and accurate monitoring of magnetic field changes in different environments is particularly important. NV centers are correlated with measurements of external magnetic fields through the Zeeman splitting of degenerate electronic states within their energy structure. The measurement of magnetic field magnitude and direction is achieved based on the extent of splitting observed in the energy structure of the NV center’s electronic states [1,2,3,4,5,6,7,8]. Moreover, its inherent long spin coherence time [9,10,11,12,13,14,15] and high sensitivity to magnetic fields allow it to simultaneously meet the requirements of high sensitivity and high spatial resolution [16,17,18] for magnetic field detection, ensures the stability and accuracy of magnetic field change measurements. Compared to traditional magnetic detection methods, magnetometers based on NV centers can significantly improve the signal-to-noise ratio, thus achieving better magnetic measurement sensitivity. Furthermore, optical means of determining magnetic resonance enable the capture of even minute signal variations and facilitate real-time data acquisition and processing. This enhances the reliability of real-time monitoring of subtle changes in magnetic field signals [19,20,21,22,23,24,25]. Building upon the inherent optical readability of diamond NV centers, a diamond NV magnetometer can be designed for rapid and precise measurement of external magnetic fields.

In recent years, there has been a significant focus on advancing the integration of NV magnetometers while maintaining their high sensitivity. This trend underscores a primary research direction in the field. Kapildeb Ambal et al. designed a differential photometry system for optically detected magnetic resonance under low photon-counting rates. The noise figure of this design reaches 4.1 μT/Hz^1/2^, allowing the continuous measurement of magnetic fields and tracking magnetic field scan rates of up to 50 μT/s [26]. However, the system still relies on bulky desktop equipment, making it difficult to meet the demands of modern portable magnetometers. To achieve the miniaturization of conventional quantum sensing platforms, Wang Xuemin et al. proposed a diamond NV magnetometer integrated with a 520 nm laser diode. The probe volume of this magnetometer was 4 × 4 × 3 cm^3^, with a magnetic field standard deviation of 75 nT and a bandwidth of 25 Hz, and this achieved a magnetic measurement noise figure of 20.77 nT/Hz^1/2^. By minimizing the volume of the light source and complex optical paths, they integrated it into the NV magnetometer system [27]. However, the dynamic range of this integrated magnetometer is only −214 μT to +214 μT. Subsequently, the United States Naval Academy utilized NV center vector magnetometers to calibrate geomagnetic anomalies, achieving a magnetic measurement noise figure of up to 200 pT/Hz^1/2^ [28]. However, the system also faced limitations in its dynamic range, effective only within a range of ±120 μT. When the magnetic field intensity exceeded the intrinsic dynamic range of NV resonance, the system would be unable to detect magnetic field changes in real-time, thus limiting its detection range in practical applications. To overcome this challenge, Wang Cao et al. employed a rapid-frequency-hopping technology to detect rapidly changing magnetic signals at 0.723 T/s [29]. Their designed NV center magnetometer extended the inherent dynamic range to 4.3 mT while maintaining a noise figure of 4.2 nT/Hz^1/2^. This achievement provides important insights for the development of integrated magnetometers with high dynamic range. Therefore, to further expand the application scope of NV magnetometers, the development of magnetometers with high dynamic range, high sensitivity, and good integration has become a key research task.

This study proposes a frequency-tracking scheme for external time-varying magnetic fields. By utilizing the resonance frequency shift caused by magnetic field changes as a feedback variable, it achieves frequency adjustment of the microwave source, thus tracking and locking the NV resonance peak. This provides a continuous measurement method for time-varying magnetic fields [30,31,32].

## 2. Principle

**Figure 1:**
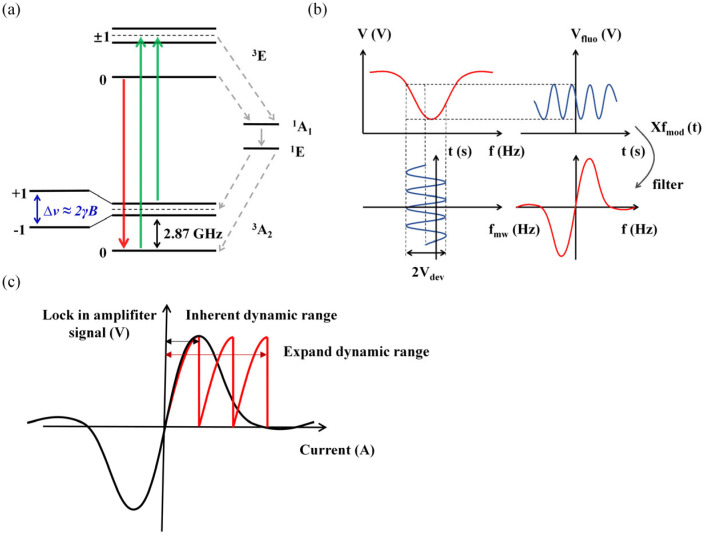
(**a**) Schematic diagram of diamond NV center energy level. The green line represents the excitation of NV centers from their ground state to the excited state induced by a 532 nm laser. The red line indicates that, after a certain duration, some NV centers undergo direct transition from the excited state to the ground state, emitting red fluorescence with a wavelength ranging from 637 to 800 nm. The gray line illustrates the pathway for NV centers to return to their ground state via the metastable states ^1^A_1_ and ^1^E; (**b**) schematic diagram of modulation–demodulation principle; (**c**) schematic diagram of frequency-tracking principle. The black arrow indicates the intrinsic dynamic range, and the red arrow indicates the extended dynamic range.

NV centers are point defects formed by the substitution of a nitrogen atom for a carbon atom in the diamond lattice, accompanied by a nearby vacancy. Carbon atoms in diamonds are arranged in a tetrahedral structure; hence, the axial direction of NV centers is constrained to $\left[111\right],\left[1\bar{1}\bar{1}\right],\left[\bar{1}\bar{1}1\right],\left[\bar{1}1\bar{1}\right]$, exhibiting C_3v_ rotational symmetry. As shown in Figure 1a, the spin triplet system consists of the m_s_ = 0 state, m_s_ = +1 state, and m_s_ = −1 state. NV centers exhibit two spin triplet states: the ground state ^3^A_2_ and the excited state ^3^E.

The NV spin state in the ground state ^3^A_2_ transitions to the excited state ^3^E under laser pumping, and then decays back to the ^3^A_2_ state through two pathways. Some NV centers directly return to the ground state from the excited state, emitting red fluorescence. Another portion of NV centers returns to the ground state through singlet states ^1^E and ^1^A_1_, which does not result in fluorescence. In the ground state, the spin triplet is degenerate for m_s_ = ±1 states in the absence of a magnetic field, while a zero-field splitting of D = 2.87 GHz exists between the m_s_ = ±1 and m_s_ = 0 states. External microwave fields can induce population transfer between the m_s_ = 0 and m_s_ = ±1 states of NV centers. For the m_s_ = ±1 states of the electron, applying an external bias magnetic field induces Zeeman splitting between the m_s_ = +1 and m_s_ = −1 energy levels, resulting in two resonance peaks [33]. The external magnetic field applied to the NV axis can be determined by measuring the frequency shift of the resonance peaks in the optically detected magnetic resonance (ODMR) spectrum. The relationship between the frequency shift induced by the Zeeman splitting and the applied external magnetic field intensity is given by Equation (1) [34].

(1) Δf=2γBcosθ

where Δf is the frequency shift, γ is the gyromagnetic ratio (taken as 2.8 × 10^10^ Hz/T), and θ is the angle between the magnetic field direction and the NV axis. Typically, the diamond NV axis is adjusted so that one of its axes aligns with the direction of the measured magnetic field. In this case, the angle θ is 0, and cosθ equals 1.

By modulating the ODMR signal with a reference signal applied at microwave frequencies using a microwave source, and subsequently performing multiplication with the reference signal followed by filtering, the demodulated signal as illustrated in Figure 1b can be obtained.

The above magnetic measurement method based on ODMR requires the calculation of the resonance peak shifts in the ODMR spectrum. However, when detecting time-varying magnetic fields, continuous scanning of the entire spectrum is required as the magnetic field changes in order to obtain the NV resonance frequency. Continuous scanning of the entire spectrum is time-consuming, as it involves monitoring non-resonant signals containing irrelevant information. Moreover, due to the lengthy scanning time, real-time detection of magnetic field changes is not feasible, which hampers the subsequent applications of NV magnetometers. Therefore, this study employs a microwave frequency modulation scheme to modulate the ODMR signal and multiply it with a reference signal, followed by filtering and demodulation. Within the linear range of the demodulated signal, the magnitude of the applied magnetic field intensity can be determined using Equation (2).

(2) $$B=\frac{V}{\gamma max\left|\frac{dv}{df}\right|}$$

Here, V represents the voltage output of the step signal, γ is the gyromagnetic ratio, and $max\left|\frac{dv}{df}\right|$ is the slope of the fitted line within the linear range of the demodulated signal. By pre-calibrating the relationship between the voltage values of the ODMR signal and the resonance frequencies, real-time monitoring of resonance point shifts in the spectrum can be achieved. However, this method is only applicable within the linear range of the demodulated signal, namely within the intrinsic dynamic range, as illustrated in Figure 1c. It cannot be used to measure magnetic fields beyond the approximately linear dynamic range interval. To enable the real-time measurement of rapidly changing external magnetic fields over a broader range, this study employs a frequency-tracking scheme. Frequency tracking is a process of monitoring real-time changes in the demodulated signal frequency and taking corresponding measures to maintain or adjust the signal frequency. In diamond NV magnetometers, frequency tracking typically refers to adjusting the microwave source frequency to match the resonance frequency changes induced by the magnetic field in the diamond sample. By continuously monitoring the resonance frequency variations induced by the magnetic field, the system is capable of dynamically adjusting the center frequency of the microwave source to keep it within the linear range of the demodulated signal, thereby extending the intrinsic dynamic range, as shown in Figure 1c.

## 3. Experimental Setup Design

**Figure 2:**
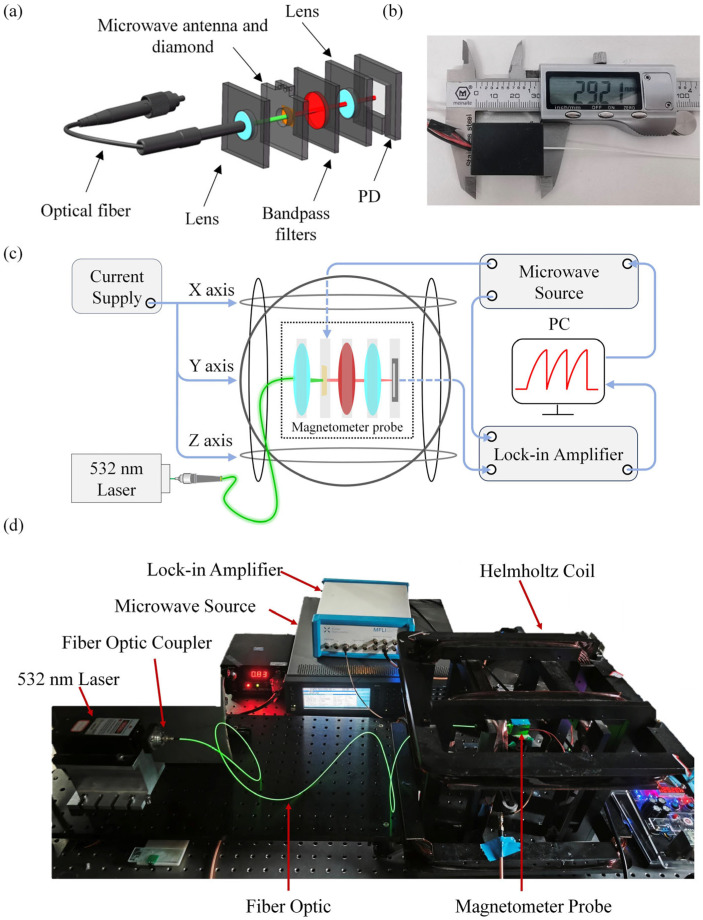
(**a**) Schematic diagram of the structure of an NV center magnetometer probe; (**b**) photograph of the magnetometer probe; (**c**) schematic diagram of the frequency tracking test system; (**d**) experimental test system physical diagram.

This study presents a portable fiber-integrated NV magnetometer. A 532 nm wavelength laser is coupled into a single-mode optical fiber and focused on the diamond surface fixed on an antenna board using a lens (SBJ30F5-21C, Changsha, China), exciting the NV centers to emit fluorescence. The red fluorescence is filtered by a bandpass filter (LBTEK, NF-8-532-SP, Changsha, China) and focused again through a lens before being collected by a photodiode (LUNAINC, PDB-C609-2, Roanoke, VA, USA) and converted into electrical signals. These signals are subsequently precisely transmitted to a lock-in amplifier for signal demodulation and processing. The demodulated signals are visualized through the software interface of the lock-in amplifier’s host computer, enabling real-time monitoring and analysis of the measured magnetic field. During this process, the microwave sweep frequency is provided by a microwave source through a circular copper wire antenna with a diameter of 1 mm. This antenna possesses omnidirectional radiation characteristics, capable of generating a uniform microwave field, while also exhibiting strong resistance to interference, ensuring the accuracy and stability of signal transmission. The fiber optic head, lens, filter, photodiode, and antenna board are all fixed within 3D-printed mounting rings, creating a closed structure that prevents external stray light from entering. Utilizing micro–nano fabrication techniques, an integrated module based on fiber optics for excitation and noise reduction can be achieved. Leveraging the efficient coupling of fiber optics and the focusing and collimating properties of self-focusing lenses, the use of traditional optical lenses in the optical path can be minimized. This greatly simplifies the optical path structure, increases the integration level of the magnetometer, and effectively reduces optical background noise in the environment. This structure allows the laser beam emitted from the laser to be focused at the center of the diamond sample. The overall structure of the magnetometer probe is shown in Figure 2a, with Figure 2b depicting the physical appearance of the probe, measuring 2.9 × 2.1 × 2 cm^3^. Combining the NV magnetometer probe with a lock-in amplifier (HF2LI, Zurich, Switzerland), microwave signal source (N5181B, Santa Clara, CA, USA), 532 nm laser (MGL-III-532-100 mW, Changchun, China), and current source (YP03-60, Changchun, China) forms a complete testing system, as shown in Figure 2c. The physical diagram of the experimental test system is shown in Figure 2d.

## 4. Experimental Details and Results

**Figure 3:**
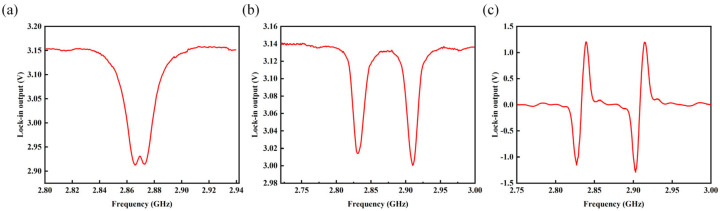
(**a**) ESR signal; (**b**) ODMR signal; (**c**) demodulated signal.

After setting up the magnetic sensing system, with the 532 nm laser controlled to output laser power of 80 mW and the microwave source set to output microwave power of 20 dBm, the obtained electron spin resonance (ESR) signal is depicted in Figure 3a. The contrast was measured to be 7.5%. By applying current from the current source to the three-axis Helmholtz coil (YP20201118-273-1, T/A:0.0016) along the X-axis, a uniform bias magnetic field is applied to the magnetometer probe. This resulted in obtaining the ODMR signal as shown in Figure 3b. The full width at half maximum (FWHM) was 13 MHz. Subsequent frequency modulation yields the demodulated signal as depicted in Figure 3c.

**Figure 4:**
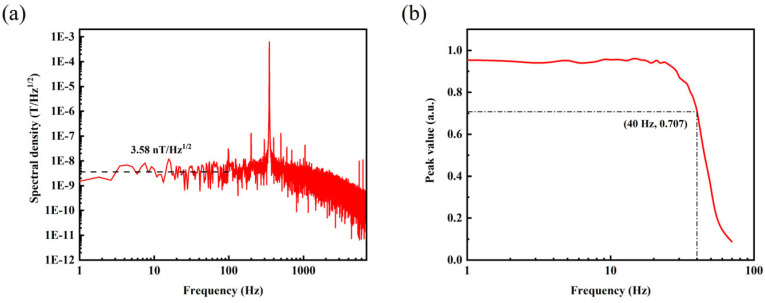
(**a**) Noise amplitude spectrum; (**b**) results of the system bandwidth.

System noise is one of the primary fundamental parameters used to measure the performance of a magnetometer. Its value represents the ability of the magnetometer to detect the smallest signal. Analyzing the amplitude spectral density is a common method currently used to calibrate the magnitude of magnetometer noise. It can reflect the output noise values of the magnetic sensing system at different frequencies. The amplitude spectral density (*ASD*) of the output signal measured in a lock-in amplifier can be converted into a magnetic noise figure using Equation (3).

(3) $$\eta =\frac{ASD}{max\left(\left|\frac{d}{df}S\right|\right)\gamma }\;$$

where ASD is obtained from the spectrum density module of the lock-in amplifier, as shown in Figure 4a. $max\left(\left|\frac{d}{df}S\right|\right)$ represents the maximum slope of the linear fit to the demodulated signal in the linear range. γ = 2.8 × 10^10^ Hz/T is the gyromagnetic ratio. The noise figure of the magnetometer obtained is 3.58 nT/Hz^1/2^.

The microwave source center frequency was fixed to the frequency corresponding to the point of maximum slope of the demodulated signal. Using a three-axis Helmholtz coil, an AC magnetic field signal with frequencies ranging from 1 Hz to 100 Hz was applied to the diamond NV magnetometer. The recorded normalized peak-to-peak amplitudes measured by the diamond NV magnetometer are depicted in Figure 4b. From these data, the system bandwidth was determined to be 40 Hz.

**Figure 5:**
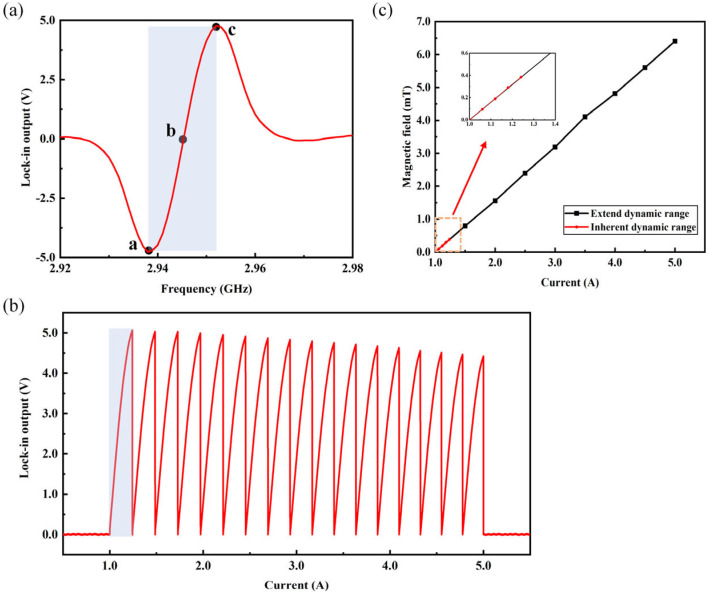
(**a**) Linear region of the demodulated signal; (**b**) schematic diagram of the frequency tracking results; (**c**) the linear relationship between the output current of the current source and the measured magnetic field value.

The method of real-time monitoring of varying magnetic fields using Equation (2) is limited by the intrinsic dynamic range of the magnetometer. As shown in Figure 5a, the region within the boxed area illustrates an approximate linear relationship between the amplitude of the demodulated signal and the microwave frequency. This linear region represents the intrinsic dynamic range of the NV magnetometer, with a slope of 1.9 mV/MHz for the fitted line. The intrinsic dynamic range of the magnetometer was approximately −384 μT to 384 μT. First, the microwave frequency was set to the frequency corresponding to the maximum slope of the linear fit to the demodulated signal. Subsequently, the current output of the power supply was gradually increased through programmatic control, causing the real-time magnetic field in the three-axis Helmholtz coil to continuously change. As the magnetic field gradually increases until the resonant frequency shifts beyond the intrinsic dynamic range, the resonant frequency point shifts from between points b and c in Figure 5a to the right of point c, exceeding the linear range. Consequently, the magnetic field conversion coefficient becomes ineffective, and during this period, the signal exhibits a decreasing trend, making it impossible to determine the magnitude of the measured magnetic field. Therefore, frequency-tracking technology is required to synchronously change the microwave frequency as the magnetic field gradually increases. This ensures that the resonant frequency point remains within the linear range. At this point, although the continuously increasing magnetic field has exceeded the magnetometer’s intrinsic measurable range, the real-time calculation of the magnetic field magnitude can still be achieved using Equation (2). This completes the extension of the intrinsic dynamic range.

Subsequently, the method is validated within the range of 1 to 5 A provided by the current source. Initially, a bias magnetic field is generated by applying a 1 A current to the three-axis Helmholtz coil. Following the completion of the microwave source frequency adjustment, the magnetic field intensity within the coil is increased by gradually increasing the output of the current source. When the current source is increased to approximately 1.24 A, the signal, which originally exhibited a decreasing trend at point c in Figure 5a, continues to gradually rise after undergoing an instantaneous change in center frequency. This ensures that the resonant frequency point remains within the linear range, thus completing the extension of the intrinsic dynamic range. Thus, it is verified that the magnetic sensing system can perform real-time magnetic field calculations within the range of 1 to 5 A provided by the current source.

Within the range from point b to point c in Figure 5a, the dynamic range is increased by approximately 17 times through the frequency tracking scheme, as shown in Figure 5b. After extending the dynamic range through frequency tracking, the relationship between the applied current values within the range of 1 to 5 A provided by the current source and the measured magnetic field magnitude by the magnetometer can be obtained as shown in Figure 5c. In Figure 5a, the same results were obtained within the range from point a to point b through this approach. Therefore, the extension of the dynamic range obtained by the magnetometer through this frequency tracking scheme is approximately 34 times greater than the intrinsic dynamic range.

The maximum tracking rate $v_{max}$ of the changing magnetic field using this frequency tracking scheme can be represented by Equation (4).

(4) $$v_{max}=\frac{\Gamma }{2t_{cycle}}$$

where Γ represents the intrinsic dynamic range of the NV center, and $t_{cycle}$ represents the period for one frequency tracking cycle. During each measurement cycle, as the magnetic field changes in real-time, the upper computer program collects and calculates the frequency offset and controls the microwave source to change the center frequency, a process taking approximately 10 ms. Within one closed-loop cycle, if the change in the magnetic field falls within half of the intrinsic dynamic range Γ/2, the system can track the resonant frequency using the frequency tracking scheme. However, if it exceeds half of the intrinsic dynamic range Γ/2, it will deviate from feedback, and the extension of the dynamic range cannot continue. Therefore, using Equation (4), the tracking rate of the system for the changing magnetic field can be calculated to be 0.038 T/s.

**Figure 6:**
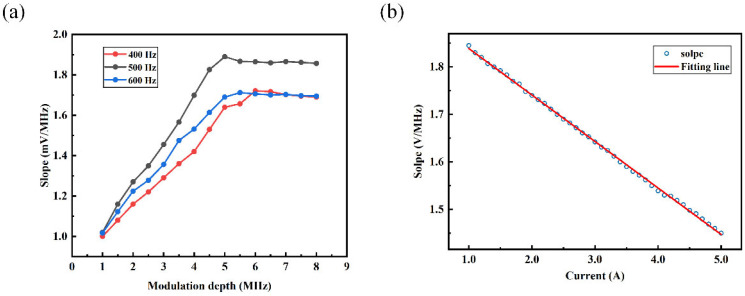
(**a**) The relationship between modulation frequency and the slope of modulation frequency offset versus demodulated signal under different modulation frequencies; (**b**) the linear relationship between the output current of the current source and the slope of the demodulated signal.

The slope of the linear fit to the demodulated signal within the linear range directly determines the frequency tracking system’s ability to extend the intrinsic dynamic range of the NV center. Therefore, it is necessary to measure the effects of modulation frequency offset and modulation frequency on the slope of the linear fit to the demodulated signal within the linear range to obtain the optimal modulation parameters. As shown in Figure 6a, during the gradual increase in modulation frequency offset, the slope initially rises and then tends to saturate. By comparing the slope values at different modulation frequencies, it can be concluded that to obtain the optimal slope value, a reference signal with V_dev_ = 5 MHz and V_mod_ = 500 Hz should be chosen.

Based on selecting the optimal modulation parameters, the slope of the linear fit to the demodulated signal within the linear range varies with the current in the range of 1 to 5 A from the current source, as shown in Figure 6b. It can be estimated that when the magnetic field reaches approximately 28.8 mT, the slope of the linear fit to the demodulated signal within the linear range approaches zero, and the slope magnitude can be considered negligible. Consequently, the magnetic field magnitude cannot be determined using Equation (2). From this, it can be concluded that due to the continuous increase in magnetic field affecting the slope of the linear fit to the demodulated signal within the linear range, there is a limit to enhancing the measurable magnetic field range through frequency tracking. Through analysis, it is determined that the theoretical limit of the extension of the magnetometer’s dynamic range is approximately 28.8 mT. Compared to the intrinsic dynamic range of the magnetometer, this represents an increase by approximately 150 times, allowing for a significant enhancement in the measurable magnetic field range of the magnetometer and stable measurement of external time-varying magnetic fields.

## 5. Conclusions

In order to realize the fast measurement of time-varying magnetic field, a frequency tracking method is proposed in this study. The time-varying magnetic field is tracked by feeding the resonant frequency shift caused by the magnetic field change back to the microwave source and continuously changing the center frequency point of the microwave source. In the experiment, the method successfully extends the dynamic range to 6.4 mT, which is equivalent to 34 times the intrinsic dynamic range of the NV center, while maintaining a tracking rate of 0.038 T/s, within the current range of 5 A that the current source can provide. Theoretical calculations show that the dynamic range of the system can be extended to 28.8 mT, which is significantly more than 150 times the intrinsic dynamic range of the NV center. In addition, by utilizing the focusing and collimation coupling characteristics of the fiber optic self-focusing lens, the fiber optic-integrated magnetometer designed in this study greatly simplifies the complex optical path structure. While maintaining portability, the system not only enhances the measurable range, but also realizes the efficient detection of continuously changing magnetic fields, which is more suitable for the complex environment of real scenarios with constantly changing magnetic fields. The research results provide an effective solution for real-time accurate monitoring of dynamic magnetic field changes, which is potentially valuable for a wide range of applications.

## Data Availability

Dataset available on request from the authors.
